# Stereo Image Matching Using Adaptive Morphological Correlation

**DOI:** 10.3390/s22239050

**Published:** 2022-11-22

**Authors:** Victor H. Diaz-Ramirez, Martin Gonzalez-Ruiz, Vitaly Kober, Rigoberto Juarez-Salazar

**Affiliations:** 1 Instituto Politécnico Nacional-CITEDI, Instituto Politécnico Nacional 1310, Tijuana 22310, BC, Mexico; 2 Department of Computer Science, CICESE, Ensenada 22860, BC, Mexico; 3 Department of Mathematics, Chelyabinsk State University, 454001 Chelyabinsk, Russia; 4 CONACYT-Instituo Politécnico Nacional, CITEDI, Instituto Politécnico Nacional 1310, Tijuana 22310, BC, Mexico

**Keywords:** stereo vision, disparity estimation, morphological correlation, locally adaptive image processing

## Abstract

A stereo matching method based on adaptive morphological correlation is presented. The point correspondences of an input pair of stereo images are determined by matching locally adaptive image windows using the suggested morphological correlation that is optimal with respect to an introduced binary dissimilarity-to-matching ratio criterion. The proposed method is capable of determining the point correspondences in homogeneous image regions and at the edges of scene objects of input stereo images with high accuracy. Furthermore, unknown correspondences of occluded and not matched points in the scene can be successfully recovered using a simple proposed post-processing. The performance of the proposed method is exhaustively tested for stereo matching in terms of objective measures using known database images. In addition, the obtained results are discussed and compared with those of two similar state-of-the-art methods.

## 1. Introduction

Stereo vision recovers three-dimensional (3-D) information about the observed scene by processing at least two images of the scene captured from different viewpoints. Stereo vision is widely used in high-impact technologies, such as robot navigation, autonomous vehicles, augmented reality and medical diagnosis, among others [1,2]. Stereo vision has many advantages over other existing 3-D technologies; for instance, simplicity and flexibility, high-rate performance, large field of view, and low cost. A fundamental task in stereo vision is disparity estimation. This task, also known as stereo matching, consists of determining the correspondence of all points in a pair of stereo images. The 3-D distribution of the scene can be retrieved from the disparity by triangulation [3].

Over the years, several approaches for stereo matching have been proposed. These approaches can be classified as local, global or hybrid [4,5]. In many applications, the local approach is preferable over the global and hybrid approaches because it is suitable for high-rate performance. In general, local methods estimate the disparity of each point of the scene by matching local windows centered at given corresponding points in each stereo image. Local methods usually perform the following steps: matching-cost computation, cost aggregation, disparity computation, post-processing and refinement [5,6,7]. The matching cost quantifies the similarity of two corresponding image points for a given disparity value. Commonly, the matching cost is computed by comparing the intensity values of two given image points. The cost aggregation reduces the uncertainty in the association of matching points. This step is usually carried out by matching adaptive windows [8,9] or adaptive weight support functions [10]. Disparity computation is performed by selecting the best aggregation cost value for each corresponding point. Post-processing recovers the disparity of occluded image points. Finally, the refinement reduces estimation errors [11,12].

Within the state-of-the-art, several methods for matching-cost computation have been suggested [13]. The absolute difference (AD), squared difference (SD) and normalized cross-correlation (NCC) are widely known intensity-based matching measures [4]. Stereo matching based on the AD, SD or NCC is computationally efficient and possesses good tolerance to image noise. However, it tends to produce incorrect disparity estimates in image regions of low texture, nonstationary intensity or that are partially occluded [6]. Alternatively, matching-cost measures based on the relative order of pixel intensities have been considered [14]. The census transform (CT) is a non-parametric technique based on the local spatial structure [7,15]. The CT maps a given image point to a binary string. Each element of this string is *true* if the intensity of a given point is higher than that of a prespecified reference point; otherwise, it is *false*. Usually, the cost aggregation in CT-based methods is computed with the Hamming distance of two resultant binary strings. The CT is more accurate than intensity-based matching methods [6]. However, it is more sensitive to image noise [16].

Several variants of the CT have been suggested to improve the stereo matching accuracy and noise robustness. A simple approach consists of replacing the intensity value of the central element of the matching window (reference point) with the mean intensity value of their neighbor elements when computing the binary string [17]. Another approach is to compute the binary string from different pairs of image points within the matching window, excluding the central point [15,16]. Recently, the use of a weighting mask in the CT matching-cost computation has been suggested [18]. In addition, a trade-off between intensity-based AD and CT has been considered [19]. This approach, known as AD-Census, has good tolerance to image noise and accuracy of disparity estimation.

Although existing local methods for stereo matching have had great success, new alternatives still need to be explored to improve their performance. For instance, in the matching-cost and cost aggregation steps, it is desirable to obtain a low cost for image points with high similarity to those belonging to the object formed at the origin of the reference window and a high cost for the remaining points. To do this, we propose a robust method for stereo matching based on adaptive morphological correlation optimized with respect to a new criterion called binary dissimilarity-to-matching ratio (BDMR). First, locally adaptive windows constructed for a reference point and a potential corresponding point in the stereo image pair are preprocessed using binary threshold decomposition. Next, the morphological correlation is computed between the two preprocessed adaptive windows for different disparity values. Finally, a disparity estimate is obtained by finding the corresponding point coordinate of the maximum correlation. In addition, we propose a simple post-processing method to recover the disparity in occluded image points.

The main contributions of this research are as follows. A binary dissimilarity-to-matching ratio (BDMR) is introduced. By minimizing the BDMR, a matching-cost measure based on adaptive morphological correlation is derived. A locally adaptive cost aggregation method for stereo matching based on morphological correlation is proposed. An efficient post-processing method for recovering the disparity of occluded and not matched stereo image points is proposed. This paper is organized as follows. Section 2 presents the proposed method for stereo image matching. Section 3 presents the results obtained with the proposed stereo matching method using images from the Middlebury stereo dataset [20,21,22]. These results are discussed and compared with those obtained with two recent existing similar methods. Finally, Section 4 presents our conclusions.

## 2. Stereo Matching with Adaptive Morphological Correlation

This section provides details of the proposed approach for stereo matching. First, we review the preliminaries of stereo vision. Secondly, we present the proposed method for image matching based on adaptive morphological correlation. Finally, we introduce the suggested approach for disparity post-processing.

### 2.1. Stereo Vision

**Figure 1:**
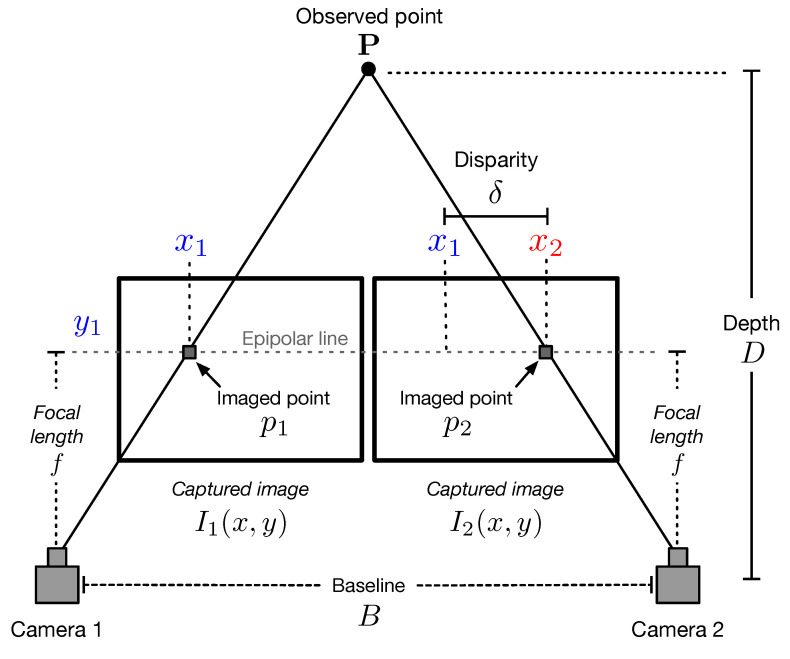
Description of a stereo imaging setup.

Consider the stereo imaging system depicted in Figure 1. A pair of cameras project a point *P* in their corresponding image planes as the points $p_{1}$ and $p_{2}$, respectively. This setup assumes that the cameras are horizontally aligned, and the captured images $I_{1}\left(x,y\right)$ and $I_{2}\left(x,y\right)$ are rectified [23,24]. Thus, the points $p_{1}$ and $p_{2}$ can be located along the horizontal epipolar line, as shown in Figure 1. The location of the points $p_{1}$ and $p_{2}$ with coordinates $\left(x_{1},y_{1}\right)$ and $\left(x_{2},y_{1}\right)$, respectively, allows us to compute the disparity as

(1) $$\delta =\left|x_{1}-x_{2}\right|.$$

The depth *D* to point *P* from the stereo baseline can be obtained as

(2) $$D=\frac{fB}{\delta },$$

where *f* is the focal length of the camera lens and *B* is the distance between the optical camera centers. It should be noted that the parameters *f* and *B* are obtained by camera calibration, and the disparity δ is determined by stereo matching.

### 2.2. Proposed Method for Stereo Matching

**Figure 2:**
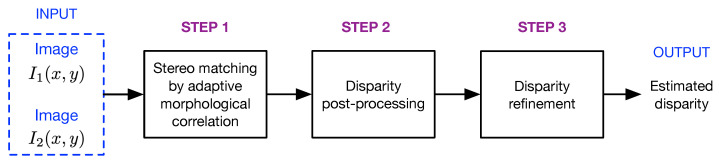
Block diagram of the proposed method for stereo image matching.

The block diagram of the proposed method is shown in Figure 2. The first step is the estimation of the disparity map from the input pair of rectified stereo images $I_{1}\left(x,y\right)$ and $I_{2}\left(x,y\right)$. Let $w_{1}\left(x,y\right)$ and $w_{2}\left(x,y\right)$ be two image windows, both of size $N_{w}\times N_{w}$, obtained from $I_{1}\left(x,y\right)$ and $I_{2}\left(x,y\right)$ at the coordinates $\left(x_{0},y_{0}\right)$, respectively. According to the theory of morphological image processing, the image window $w_{i}\left(x,y\right)$ can be represented by the binary threshold decomposition in a given range as [25,26,27]

(3) $${\tilde{w}}_{i}\left(x,y\right)=\sum_{q=q_{0}}^{q_{N}}b_{i,q}\left(x,y\right),$$

where $q_{0}=\min\left\{w_{i}\left(x,y\right)\right\}$, $q_{N}=\max\left\{w_{i}\left(x,y\right)\right\}$, and

(4) $$b_{i,q}\left(x,y\right)=\left\{\begin{array}{cc} 1, & \mathrm{if}\;w_{i}\left(x,y\right)\geq q,\\ 0, & \mathrm{otherwise}, \end{array}\right.$$

is a binary image of $w_{i}\left(x,y\right)$ for the *q*-th intensity value. Note that if $q_{0}=1$ then $w_{i}\left(x,y\right)={\tilde{w}}_{i}\left(x,y\right)$.

Now, assuming the horizontal epipolar constraint, we introduce the binary dissimilarity-to-matching ratio (BDMR) as follows:

(5) $$BDMR\left(\tau \right)=\frac{D(\tau )}{M(\tau )}=\frac{\sum_{q=q_{0}}^{q_{N}}\sum_{x=1}^{N_{w}}\sum_{y=1}^{N_{w}}\left|b_{1,q}\left(x,y\right)-b_{2,q}\left(x-\tau ,y\right)\right|}{\sum_{q=q_{0}}^{q_{N}}\sum_{x=1}^{N_{w}}\sum_{y=1}^{N_{w}}\left|b_{1,q}\left(x,y\right)+b_{2,q}\left(x-\tau ,y\right)-1\right|},$$

where the denominator M(τ) is a point-wise binary matching measure between $w_{1}\left(x,y\right)$ and $w_{2}\left(x-\tau ,y\right)$. The numerator D(τ) quantifies the binary dissimilarity of $w_{1}\left(x,y\right)$ and $w_{2}\left(x-\tau ,y\right)$. Note that the BDMR produces zero when $w_{1}\left(x,y\right)$ and $w_{2}\left(x-\tau ,y\right)$ are identical and infinity when there are no matches. We want to derive a matching-cost measure by minimization of the BDMR. Based on the properties of the absolute value, Equation (5) can be rewritten as

(6) $$BDMR\left(\tau \right)=\frac{\sum_{q=q_{0}}^{q_{N}}\sum_{x=1}^{N_{w}}\sum_{y=1}^{N_{w}}\left[b_{1,q}\left(x,y\right)+b_{2,q}\left(x-\tau ,y\right)-2MIN\left\{b_{1,q}\left(x,y\right),b_{2,q}\left(x-\tau ,y\right)\right\}\right]}{\sum_{q=q_{0}}^{q_{N}}\sum_{x=1}^{N_{w}}\sum_{y=1}^{N_{w}}\left[1+b_{1,q}\left(x,y\right)+b_{2,q}\left(x-\tau ,y\right)-2MIN\left\{b_{1,q}\left(x,y\right)+b_{2,q}\left(x-\tau ,y\right),1\right\}\right]}.$$

Note that the summation terms in Equation (6) can be calculated as

(7) $$\begin{array}{cc} \mu_{b_{1}} & =\frac{1}{QN_{w}^{2}}\sum_{q=q_{0}}^{q_{N}}\sum_{x=1}^{N_{w}}\sum_{y=1}^{N_{w}}b_{1,q}\left(x,y\right),\\ \mu_{b_{2}}\left(\tau \right) & =\frac{1}{QN_{w}^{2}}\sum_{q=q_{0}}^{q_{N}}\sum_{x=1}^{N_{w}}\sum_{y=1}^{N_{w}}b_{2,q}\left(x-\tau ,y\right), \end{array}$$

where $Q=(q_{N}-q_{0})$ is the number of quantization levels in the binary threshold decomposition. Moreover, by considering that

(8) $$MIN\left\{b_{1,q}\left(x,y\right)+b_{2,q}\left(x,y\right),1\right\}=MAX\left\{b_{1,q}\left(x,y\right),b_{2,q}\left(x,y\right)\right\},$$

Equation (6) can be rewritten as

(9) $$BDMR\left(\tau \right)=\frac{\mu_{b_{1}}+\mu_{b_{2}}\left(\tau \right)-\frac{2}{QN_{w}^{2}}\sum_{q=q_{0}}^{q_{N}}\sum_{x=1}^{N_{w}}\sum_{y=1}^{N_{w}}MIN\left\{b_{1,q}\left(x,y\right),b_{2,q}\left(x-\tau ,y\right)\right\}}{1+\mu_{b_{1}}+\mu_{b_{2}}\left(\tau \right)-\frac{2}{QN_{w}^{2}}\sum_{q=q_{0}}^{q_{N}}\sum_{x=1}^{N_{w}}\sum_{y=1}^{N_{w}}MAX\left\{b_{1,q}\left(x,y\right),b_{2,q}\left(x-\tau ,y\right)\right\}}.$$

The minimum value of Equation (9) is obtained by maximizing

(10) $$C\left(\tau \right)=\frac{\sum_{q=q_{0}}^{q_{N}}\sum_{x=1}^{N_{w}}\sum_{y=1}^{N_{w}}MIN\left\{b_{1,q}\left(x,y\right),b_{2,q}\left(x-\tau ,y\right)\right\}}{\frac{1}{QN_{w}^{2}}+\sum_{q=q_{0}}^{q_{N}}\sum_{x=1}^{N_{w}}\sum_{y=1}^{N_{w}}MAX\left\{b_{1,q}\left(x,y\right),b_{2,q}\left(x-\tau ,y\right)\right\}},$$

where the term $1\;/\;QN_{w}^{2}$ is added to the denominator to avoid singularities. Now, by interchanging the order of summations and considering [25,27]

(11) $$\begin{array}{cc} \sum_{q=q_{0}}^{q_{N}}MIN\left\{b_{1,q}\left(x,y\right),b_{2,q}\left(x,y\right)\right\} & =MIN\left\{w_{1}\left(x,y\right),w_{2}\left(x,y\right)\right\},\\ \sum_{q=q_{0}}^{q_{N}}MAX\left\{b_{1,q}\left(x,y\right),b_{2,q}\left(x,y\right)\right\} & =MAX\left\{w_{1}\left(x,y\right),w_{2}\left(x,y\right)\right\}, \end{array}$$

Equation (10) can be rewritten as

(12) $$C\left(\tau \right)=\frac{\sum_{x=1}^{N_{w}}\sum_{y=1}^{N_{w}}MIN\left\{\sum_{q=q_{0}}^{q_{N}}b_{1,q}\left(x,y\right),\sum_{q=q_{0}}^{q_{N}}b_{2,q}\left(x-\tau ,y\right)\right\}}{\frac{1}{QN_{w}^{2}}+\sum_{x=1}^{N_{w}}\sum_{y=1}^{N_{w}}MAX\left\{\sum_{q=q_{0}}^{q_{N}}b_{1,q}\left(x,y\right),\sum_{q=q_{0}}^{q_{N}}b_{2,q}\left(x-\tau ,y\right)\right\}}.$$

Equation (12) is a nonlinear correlation that minimizes the BDMR when the maximum correlation value is reached. For the problem of stereo matching, the maximum value of Equation (12) should occur in the coordinate τ=δ; that is, at the location where the sliding window $w_{2}\left(x-\tau ,y\right)$ matches the reference window $w_{1}\left(x,y\right)$. To improve the accuracy and robustness of stereo matching using Equation (12), the values $\left\{q_{0},q_{N}\right\}$ can be chosen to properly describe the implicit object formed at the origin $\left(x_{0},y_{0}\right)$ of the window $w_{i}\left(x,y\right)$, identified as the target. Thus, the values $\left\{q_{0},q_{N}\right\}$ can be specified as

(13) $$\begin{array}{cc} q_{0} & =w_{i}\left(x_{0},y_{0}\right)-\epsilon_{v}\sigma_{w_{i}},\\ q_{N} & =w_{i}\left(x_{0},y_{0}\right)+\epsilon_{v}\sigma_{w_{i}}, \end{array}$$

where $\sigma_{w_{i}}$ is the standard deviation of $w_{i}\left(x,y\right)$ with respect to $w_{i}\left(x_{0},y_{0}\right)$ and $\epsilon_{v}$ is a dispersion parameter. Thus, Equation (12) can be adapted to each point of the pair of stereo images as

(14) $$C\left(\tau \right)=\frac{\sum_{x=1}^{N_{w}}\sum_{y=1}^{N_{w}}MIN\left\{{\tilde{w}}_{1}\left(x,y\right),{\tilde{w}}_{2}\left(x-\tau ,y\right)\right\}}{\frac{1}{QN_{w}^{2}}+\sum_{x=1}^{N_{w}}\sum_{y=1}^{N_{w}}MAX\left\{{\tilde{w}}_{1}\left(x,y\right),{\tilde{w}}_{2}\left(x-\tau ,y\right)\right\}},$$

where

(15) $$\begin{array}{cc} {\tilde{w}}_{1}\left(x,y\right) & =\sum_{q=1}^{Q}b_{1,(q_{0}+q\Delta_{q})}\left(x,y\right),\\ {\tilde{w}}_{2}\left(x-\tau ,y\right) & =\sum_{q=1}^{Q}b_{2,(q_{0}+q\Delta_{q})}\left(x-\tau ,y\right), \end{array}$$

are preprocessed image windows of $I_{1}\left(x,y\right)$ and $I_{2}\left(x,y\right)$, respectively, using adaptive binary threshold decomposition, with a quantization step as

(16) $$\Delta_{q}=\frac{2\epsilon_{v}\sigma_{w_{1}}}{Q}.$$

**Figure 3:**
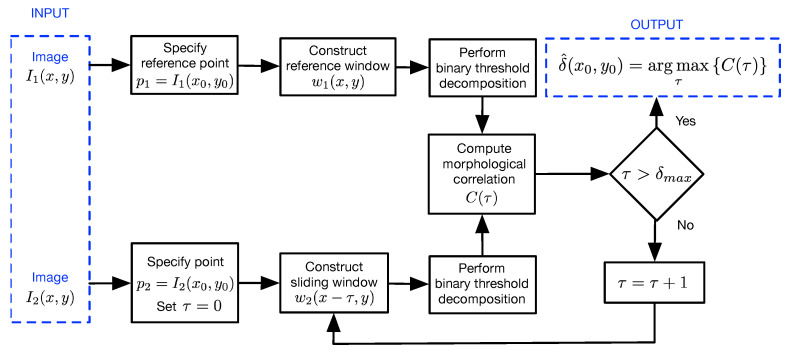
Block diagram of the proposed method for stereo matching based on adaptive morphological correlation.

To perform stereo matching using Equations (14)–(16), consider a reference point $p_{1}$ with coordinates $\left(x_{0},y_{0}\right)$ in the image $I_{1}\left(x,y\right)$. The corresponding point $p_{2}$ in image $I_{2}\left(x,y\right)$ can be detected and located as depicted in the block diagram shown in Figure 3. First, a reference window $w_{1}\left(x,y\right)$ with origin at the point $p_{1}$ and size of $N_{w}\times N_{w}$ is constructed from $I_{1}\left(x,y\right)$, where $N_{w}=2s+1$ and *s* are computed adaptively as

(17) $$s=\left(s_{0}-1\right)\exp\left(-\beta \frac{I_{1}\left(x_{0},y_{0}\right)}{\sigma_{s_{0}}^{2}}\right),$$

where β is a scalar, $s_{0}$ is a prespecified parameter defining the maximum allowable window size and $\sigma_{s_{0}}^{2}$ is the standard deviation of the intensity values of the points within the reference window with a maximum size of $\left(2s_{0}+1\right)\times \left(2s_{0}+1\right)$ with respect to $p_{1}$. Then, a sliding window $w_{2}\left(x-\tau ,y\right):\tau \in \left[0,\delta_{max}\right]$, with a size of $N_{w}\times N_{w}$ is constructed from $I_{2}\left(x,y\right)$. Note that $w_{2}\left(x-\tau ,y\right)$ is shifted along the horizontal epipolar line of $I_{2}\left(x,y\right)$. Afterward, $w_{1}\left(x,y\right)$ and $w_{2}\left(x-\tau ,y\right)$ are preprocessed by binary threshold decomposition as described in Equation (15). Next, the adaptive morphological correlation is given in Equations (14)–(16) is computed for all values of τ. Finally, a disparity estimate is obtained as

(18) $$\delta \left(x_{0},y_{0}\right)=\underset{\tau }{\arg\max}\left\{C(\tau )\right\}.$$

The disparity maps $\delta_{1}\left(x,y\right)$ and $\delta_{2}\left(x,y\right)$ can be obtained by applying the proposed method to all points of the stereo images $I_{1}\left(x,y\right)$ and $I_{2}\left(x,y\right)$.

### 2.3. Disparity Post-Processing

The estimated disparity maps $\delta_{1}\left(x,y\right)$ and $\delta_{2}\left(x,y\right)$, can be verified as

(19) $$m_{k}\left(x,y\right)=\left\{\begin{array}{cc} 1, & \mathrm{if}\;\left|\delta_{k}\left(x,y\right)-\delta_{l}\left(x-\delta_{k}\left(x,y\right),y\right)\right|\leq \epsilon_{\delta },\\ 0, & \mathrm{otherwise}, \end{array}\right.$$

where $\epsilon_{\delta }$ is a tolerance parameter, $k=\left\{1,2\right\}$ and $l=\left\{\begin{array}{cc} k+1, & \mathrm{if}\;k=1\\ k-1, & \mathrm{if}\;k=2. \end{array}\right.$

Note that a value of $m_{k}\left(x,y\right)=1$ in Equation (19) indicates a verified estimated disparity, whereas a value of $m_{k}\left(x,y\right)=0$ denotes an incorrectly estimated disparity caused by an occlusion or any other perturbation. Let $T=\left\{\left(x_{T},y_{T}\right):m_{i}\left(x_{T},y_{T}\right)=1\right\}$ be the set of coordinates of all verified estimated disparities and $F=\left\{\left(x_{F},y_{F}\right):m_{i}\left(x_{F},y_{F}\right)=0\right\}$ be the set of coordinates of all incorrectly estimated disparities. A desirable post-processing method requires replacing the incorrectly estimated disparity value $\delta_{i}\left(x_{F},y_{F}\right)$ with verified disparity values from the set $\left\{\delta_{i}\left(x_{T},y_{T}\right)\right\}$. In this context, we consider the prior probability that a verified estimated disparity at arbitrary coordinates (x,y) can replace the incorrect disparity at the coordinates $\left(x_{F},y_{F}\right)$, which is given by

(20) $$P\left(x,y\right)=\frac{1}{\sigma_{1}\sqrt{2\pi }}\exp\left[-\frac{{\left(x-x_{F}\right)}^{2}+{\left(y-y_{F}\right)}^{2}}{2\sigma_{1}^{2}}\right]:\left(x,y\right)\in T,$$

where a normal distribution with variance $\sigma_{1}^{2}$ is assumed. Furthermore, the probability density function that an image point with intensity value $I_{i}\left(x,y\right)$ has a similar disparity as that expected at the coordinates $\left(x_{F},y_{F}\right)$, can be given by

(21) $$P\left(I_{i}\left(x,y\right)|\delta_{i}\left(x_{F},y_{F}\right)\right)=\frac{1}{\sigma_{2}^{2}\sqrt{2\pi }}\exp\left[-\frac{{\left(I_{i}\left(x,y\right)-I_{i}\left(x_{F},y_{F}\right)\right)}^{2}}{2\sigma_{2}^{2}}\right]:\left(x,y\right)\in T,$$

where $\sigma_{2}^{2}$ is the variance of the target’s intensity values. According to Bayesian theory, the posterior probability that an image point with disparity δ(x,y) and intensity $I_{i}\left(x,y\right)$ can replace unknown disparity $\delta_{i}\left(x_{F},y_{F}\right)$ given that $I_{i}\left(x_{F},y_{F}\right)$ is the intensity of $I_{i}\left(x,y\right)$ at the coordinates $\left(x_{F},y_{F}\right)$ is given as

(22) $$P\left(\delta_{i}\left(x,y\right)|I\left(x_{F},y_{F}\right)\right)=\frac{P\left(I_{i}\left(x,y\right)|\delta_{i}\left(x_{F},y_{F}\right)\right)P\left(x,y\right)}{P\left(I_{i}\left(x,y\right)\right)},$$

where $P\left(I_{i}\left(x,y\right)\right)$ is the prior probability density function of the intensity of $I_{i}\left(x,y\right)$. As a result, the coordinates $(x_{T},y_{T})\in T$ of the disparity $\delta (x_{T},y_{T})$ with the highest probability corresponds to the unknown disparity $\delta (x_{F},y_{F})$, and can be obtained as

(23) $$\left({\hat{x}}_{T},{\hat{y}}_{T}\right)=\underset{(x,y)\in T}{\arg\max}\left\{P\left(\delta_{i}\left(x,y\right)\right)|I_{i}\left(x_{F},y_{F}\right)\right\}.$$

By substituting Equations (20) and (21) into Equation (23), and by applying the logarithm function, we get

(24) $$\left({\hat{x}}_{T},{\hat{y}}_{T}\right)=\underset{(x,y)\in T}{\arg\min}\left\{\frac{{\left(x-x_{F}\right)}^{2}+{\left(y-y_{F}\right)}^{2}}{2\sigma_{1}^{2}}+\frac{{\left(I_{i}\left(x,y\right)-I_{i}\left(x_{F},y_{F}\right)\right)}^{2}}{2\sigma_{2}^{2}}\right\},$$

where $\delta_{i}\left({\hat{x}}_{T},{\hat{y}}_{T}\right)$ is an estimate of the incorrect disparity $\delta_{i}\left(x_{F},y_{F}\right)$. Thus, by applying the estimator given in Equation (24) to all elements of the set *F*, one can obtain the improved post-processed disparity maps $\delta_{1}\left(x,y\right)$ and $\delta_{2}\left(x,y\right)$.

## 3. Results

This section presents the results obtained with the proposed approach for stereo matching using images from the Middlebury stereo dataset [20,21,22]. The results are discussed and compared with those obtained with two recent variants of the CT, namely, the improved weighted census transform (IWCT) [18] and the improved AD-Census (AD-C) algorithm [19]. The accuracy of disparity estimation by the proposed, IWCT and AD-C methods is quantified in terms of the bad-matched pixels (BMP) and root mean squared (RMS) error between estimated and ground truth disparities. For the BMP measure, we set the tolerance of $\epsilon_{\delta }=2$. First, we quantify the performance of the proposed and considered methods for disparity estimation of non-occluded regions in input stereo images. Next, we evaluate the performance of the suggested disparity post-processing method. Additionally, we show refined disparity maps obtained with the proposed approach using a generic refinement method. Finally, we present the statistical performance results of the proposed and considered methods for stereo matching with twenty-five images from the Middlebury stereo dataset.

**Figure 4:**
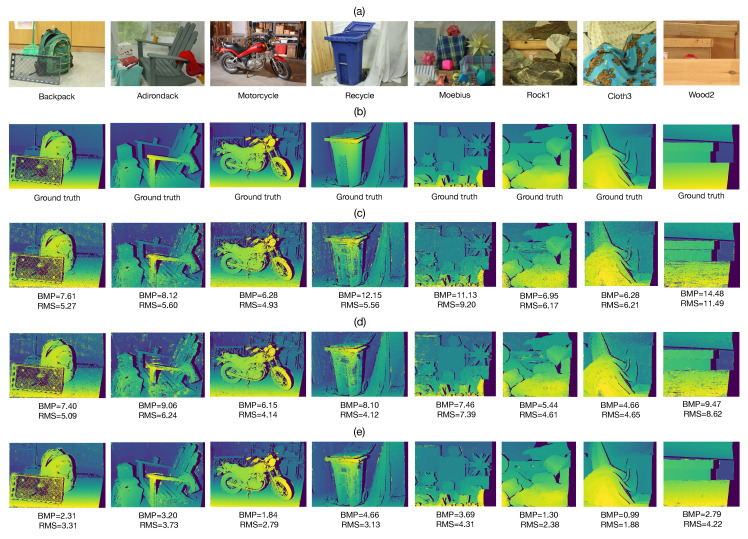
Disparity estimation results for non-occluded image regions. (**a**) Right input stereo image. (**b**) Ground truth disparity map of non-occluded image regions. Estimated disparity maps of non-occluded image regions, obtained with: (**c**) IWCT, (**d**) AD-C and (**e**) Proposed method.

The proposed method, IWCT and AD-C were implemented using the Python 3.10.7 language on a personal computer with an Intel Core I5 2.4 GHz processor, 16 GB of RAM and Linux Ubuntu 20.04 operating system. Figure 4a shows the right image from eight different stereo image pairs from the dataset. The window size for all tested methods is $N_{w}\times N_{w}$, where $N_{w}=2s_{0}+1$ and $s_{0}=6$. For the proposed method we set Q=31, $\epsilon_{v}=1.5$ and $\beta =2.5s_{0}$. Figure 4b shows the ground truth disparities of non-occluded regions of the input images shown in Figure 4a. The non-occluded regions are obtained by applying the verification method given in Equation (19) to the ground truth disparities provided by the dataset. The estimated disparity maps obtained with the IWCT, AD-C and proposed method are presented in Figure 4c–e, respectively. Notice that the proposed method produces the lowest values of BMP and RMS measures in all cases compared to those obtained with the IWCT and AD-C methods. The proposed method is able to estimate the disparity in homogeneous regions with high accuracy. This feature is obtained when the specified number *Q* of quantification values for the binary threshold decomposition is sufficiently large (Q>8). Furthermore, it can be seen that the proposed method is also able to correctly estimate the disparity at the edges of the objects in the scene. This feature is due to the dynamic adaptation of the sliding windows employed for point matching given in Equation (17). On the other hand, the IWCT method produces the worst results of all tested methods. This approach yields many incorrectly estimated disparity values in homogeneous image regions. Note that the test images shown in Figure 4a present several challenges for stereo matching, such as image regions with little texture, partial occlusions, nonstationary intensity changes, objects with sharp edges and abrupt disparity variations. According to the obtained results shown in Figure 4c–e, the proposed method adapts better to challenging situations than the other tested methods. However, the lack of texture in image regions larger than the search space of the algorithm causes the matching method to be unable to determine the point correspondences. For instance, this can be seen in the central area of the Recycle image. The AD-C algorithm yields good results in the majority of the performed tests. This algorithm can estimate the disparity values at the edges of the scene objects very well. However, its performance is lower than that of the proposed method.

**Figure 5:**
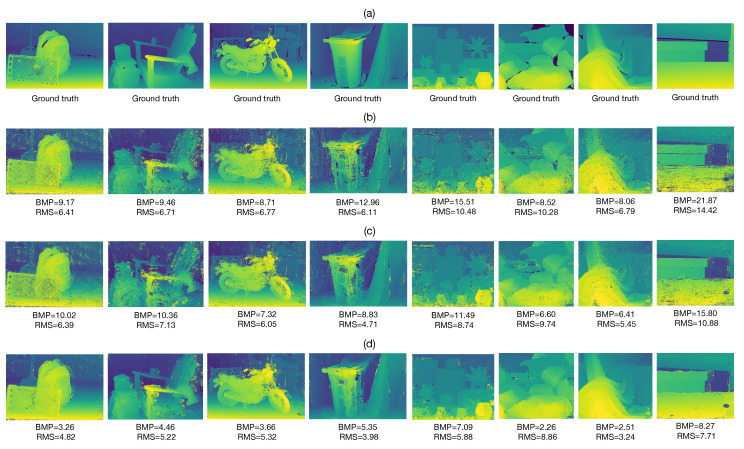
Results of disparity post-processing using the suggested method. (**a**) Ground truth disparity maps. Post-processed disparity maps with the suggested method, obtained with: (**b**) IWCT, (**c**) AD-C and (**d**) Proposed method.

Afterward, we evaluated the performance of the post-processing method described in Section 2.3. We applied the suggested post-processing to the estimated disparity maps obtained with the IWCT, AD-C and proposed method, see Figure 4c–e. The resultant post-processed disparity maps are presented in Figure 5b–d. It can be seen that the suggested post-processing is successful in retrieving the unknown disparity values in occluded regions of the input stereo images. Furthermore, it can also retrieve several incorrectly estimated disparity values in homogeneous image regions, which were not verified by Equation (19). Additionally, Figure 5b–d presents the BMP and RMS values of all tested methods between the estimated and ground truth disparities shown in Figure 5a. The IWCT and AD-C methods yield higher BMP and RMS values in comparison with those obtained with the proposed method. The AD-C algorithm yields slightly better performance than the IWCT. However, the post-processed disparity maps obtained with the proposed method yielding the best results of all the tested methods. It is worth mentioning that the large occluded regions on the right side of the estimated disparity maps shown in Figure 4c–e were correctly recovered by the suggested post-processing in all tested methods. However, note that the post-processing was unable to recover the disparity values in the wood knot shown in the Wood2 image. This is because the verified disparity values in the vicinity of this region are associated with image points with intensity values that are significantly different from those of the wood knot.

**Figure 6:**
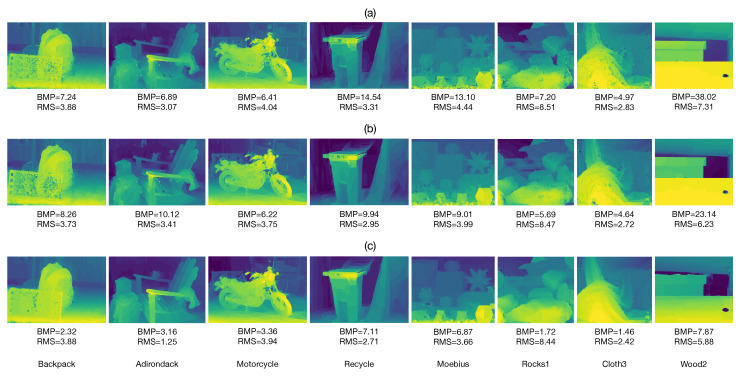
Refined disparity maps after applying the suggested post-processing method, obtained with: (**a**) IWCT, (**b**) AD-C and (**c**) Proposed method.

Now, the post-processed disparity maps shown in Figure 5b–d, were refined by applying the well-known weighted least-squares filter [28]. The refined disparity maps are shown in Figure 6. Note that the refinement significantly reduces anomalous disparity errors for all tested methods. The refined disparity maps using the proposed adaptive morphological correlation approach produce the best results of all tested methods in terms of the BMP and RMS measures. Furthermore, we see that the refined disparity maps using the IWCT and AD-C methods of images, such as Adirondack, Recycle and Rocks1, contain very noticeable artifacts, while the refined disparity maps using the proposed approach contain fewer artifacts. This result is expected because any refinement method performs better when the input disparity map contains fewer incorrect disparity estimates, such as those obtained with the proposed approach.

**Figure 7:**
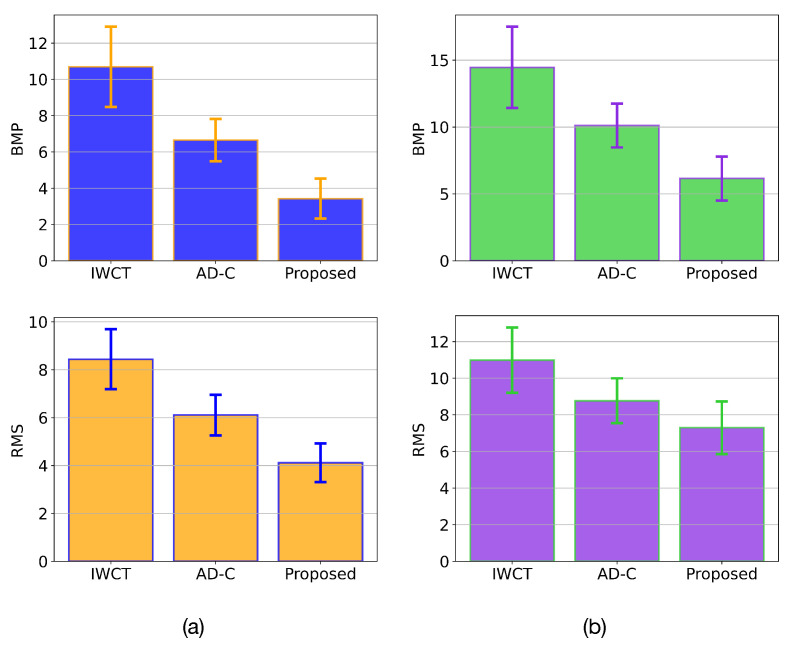
Statistical performance results in terms of BMP and RMS measurements in twenty-five stereo images of the evaluated methods. (**a**) Disparity estimation results in non-occluded image regions. (**b**) Statistical performance of the suggested method of disparity post-processing.

Finally, we compare the statistical performance of the proposed method, IWCT and AD-C, in terms of both BMP and RMS measures. In this experiment, we estimated the disparity map of twenty-five different stereo images from the Middlebury stereo dataset using each of the considered stereo matching methods. The mean value and standard deviation of the BMP and RMS measurements were computed for each tested method. The results are presented in Figure 7 and Table 1. Figure 7a shows the statistical results for all tested stereo matching methods considering only the non-occluded regions of the input stereo images. Note that the proposed approach yields the best results of all tested methods. In contrast, the IWCT produces the worst results. This low performance is because the IWCT produces many wrong disparity estimates in homogeneous image regions, as shown in Figure 4c. The AD-C algorithm yields good statistical results in general terms. The AD-C approach produces fewer incorrect disparity estimates in homogeneous image regions than the IWCT. Additionally, it correctly estimates the disparity values at the edges of the objects present in the scene. However, the performance of both AD-C and IWCT methods is lower than that of the proposed approach.

Figure 7b presents the statistical results of the post-processed disparity maps using the suggested approach. It should be noted that the reference for computing the BMP and RMS measures consists of the ground truth disparities provided by the dataset, see Figure 5a. Note that the proposed approach yields the best results, whereas IWCT yields the worst results. The AD-C algorithm produces acceptable results in general terms. The results shown in Figure 7a and Table 1 confirm that the proposed method based on adaptive morphological correlation is effective and robust for stereo image matching. Additionally, the results presented in Figure 7b and Table 1 indicate that the suggested post-processing method is successful in retrieving the disparity values in occluded image regions.

## 4. Conclusions

An accurate and robust method for stereo image matching based on adaptive morphological correlation was presented. The correspondence of non-occluded points in a pair of rectified stereo images was accurately determined by matching locally adaptive image windows using the suggested morphological correlation operation, which is optimal with respect to the new, introduced criterion called binary-to-dissimilarity ratio. In addition, a simple disparity post-processing method for recovering point correspondences of occluded points was suggested. The performance of the proposed method for stereo matching was exhaustively tested in terms of the mean absolute error and peak signal-to-noise ratio objective measures using images of the well-known Middlebury stereo dataset. The obtained results were discussed and compared with two recent state-of-the-art methods based on the census transform. According to the performed experiments and obtained results, the proposed method for stereo matching outperformed the existing tested methods in terms of the considered performance measures. Additionally, the obtained results confirmed that the suggested post-processing method allowed the disparity values of partially occluded image points to be successfully recovered.

## Figures and Tables

**Table 1:** Statistical results in terms of BMP and RMS for stereo matching of non-occluded points and evaluation of the proposed post-processing method.

	Stereo Matching Non-Occluded Points				Proposed Post-Processing			
	BMP		RMS		BMP		RMS	
**Method**	**Mean**	**St. Dev.**	**Mean**	**St. Dev.**	**Mean**	**St. Dev.**	**Mean**	**St. Dev.**
IWCT	10.69	4.41	8.44	2.51	14.46	6.07	10.99	3.57
AD-C	6.65	2.33	6.10	1.69	10.11	3.28	8.77	2.45
Proposed	3.42	2.20	4.11	1.62	6.15	3.28	7.29	2.87

## Data Availability

Publicly available datasets were analyzed in this study. This data can be found here: vision.middlebury.edu (accessed on 14 October 2022).
